# Comparative effectiveness of guided internet-based stress management training versus established in-person group training in employees – study protocol for a pragmatic, randomized, non-inferiority trial

**DOI:** 10.1186/s12889-021-12229-y

**Published:** 2021-11-27

**Authors:** Leif Boß, Peter Angerer, Nico Dragano, David Ebert, Miriam Engels, Elena Heber, Rebekka Kuhlmann, Sascha Ruhle, Christian Schwens, Ines Catharina Wulf, Dirk Lehr

**Affiliations:** 1 Department of Health Psychology and Applied Biological Psychology, Institute of Psychology, Leuphana University Lüneburg, Lüneburg, Germany; 2 Institute of Occupational, Social and Environmental Medicine, Centre for Health and Society, Medical Faculty and University Hospital, Heinrich-Heine-University Düsseldorf, Düsseldorf, Germany; 3 Institute of Medical Sociology, Centre for Health and Society, Medical Faculty and University Hospital, Heinrich-Heine-University Düsseldorf, Düsseldorf, Germany; 4 Department of Sport and Health Sciences, Technical University of Munich, Munich, Germany; 5 GET.ON Institut für Online Gesundheitstrainings GmbH, Hamburg, Germany; 6 Chair of Business Administration, in particular Work, Human Resource Management and Organization Studies, Faculty of Business Administration and Economics, Heinrich-Heine-University Düsseldorf, Düsseldorf, Germany; 7 Endowed Chair for Interdisciplinary Management Science, School of Management, Economics and Social Sciences, University of Cologne, Cologne, Germany

**Keywords:** Web-based intervention, Stress management, Occupational health, E-mental health, Randomized controlled trial, Non-inferiority trial

## Abstract

**Background:**

Occupational stress is a major public health challenge that requires a variety of evidence-based preventative approaches to increase their reach within the working population. Behavioral stress management interventions are considered an established approach for occupational stress prevention. Both in-person group-based stress management training (gSMT) and individual Internet-based training (iSMT) have been shown to be effective at reducing stress in employees. However, there remains a lack of evidence on the comparative efficacy of the newer digital format compared to well-established, in-person, group-based training. This study aims (1) to directly compare an evidence-based iSMT with an established gSMT on stress in employees, (2) to analyze the two conditions from a cost perspective, and (3) to explore moderators of the comparative efficacy.

**Methods:**

In a randomized, controlled, non-inferiority trial employees from the general working population will be allocated to iSMT or gSMT. The primary outcome will be perceived stress, assessed using the Perceived Stress Scale, three months after randomization. The non-inferiority margin for the primary outcome measure will be set at 2 points (Cohen’s d = 0.29). This trial will also compare the two interventions from a health economics perspective, and conduct explorative analyses to identify potential effect moderators.

**Discussion:**

To reach a larger proportion of the working population, well-established gSMT should be complemented with interventions that fit today’s society’s increasingly digital lifestyle. The current trial will provide evidence supporting the responsible implementation of Internet-based stress management training if the digital format proves to at least be non-inferior to established group-based training. Additional explorative moderator analyses may guide future practices to aid in matching select programs with select users.

**Trial registration:**

German Register of Clinical Studies (DRKS): DRKS00024892, date of registration: 2021-04-09.

Protocol version: 02, 16-10-2021.

**Supplementary Information:**

The online version contains supplementary material available at 10.1186/s12889-021-12229-y.

## Background

There is meta-analytic evidence documenting an association between work-related stress and an increased risk of developing severe disorders like depression [1], musculoskeletal conditions [2] and coronary artery disease [3–5]. Work-related stress also accounts for considerable societal costs, the majority due to productivity losses [6, 7].

In recent decades, numerous interventions have been developed and evaluated to protect workers from stress and its adverse health effects [8, 9]. Among the various measures developed to reduce stress, strong evidence exists suggesting beneficial effects from stress management training (SMT) [10–13]. Typically, SMT is offered as group stress management training (gSMT), mostly led by external trainers [10, 11]. At larger companies, specialized professional units for health and safety usually offer such mental health programs to the employees as in-house training programs. This requires organizational and financial resources, as well as the motivation of enough employees to participate in group training [14]. However, in the United States, for example, about half of all employees currently work at a small or medium-sized company [15], and similar figures exist for the European Union [16]. Furthermore, employers may not always be aware of the business benefits of employee health interventions [17]. Additionally, some practical issues specific to small businesses may hamper the provision of gSMT. Examples of this are the non-availability of service providers in rural areas [14] and inadequate numbers of employees to justify in-house group training at small businesses. One alternative is joint group training involving employees from multiple companies. Potential disadvantages of this are that such training might require extra travel-time to reach the venue and a loss in autonomy due to externally-determined time schedules.

To overcome some of the barriers of in-person group stress management training, Internet-based interventions have been advocated as an alternative [18]. They fit with an increasingly digital lifestyle and employees can use Internet-based stress management training (iSMT) at any time and place, at their own pace, and without the time and costs required for travel. Moreover, anonymous participation is possible, if participants prefer to not disclose struggling with stress to their colleagues and/or employer. Studies have shown that iSMT can be effective in different employee groups with, on average, moderate-to-large effects, in terms of reducing stress [19, 20]. In addition, meta-analyses have revealed small to moderate effects on the symptoms of depression and anxiety [20, 21], large effects on insomnia [20] and even effects on work productivity [22]. ISMT appears to work, either with or without additional human support (i.e., guided by written feedback on training assignments), but guided interventions generate larger mean effects than unguided programs [19]. Moreover, initial studies have shown that iSMT can be cost-effective for both employers [23] and society [24], relative to non-active control conditions.

However, the majority of previously published studies compared interventions to non-active control conditions [19]. These results are helpful for deciding whether iSMT is effective in principle. For policy and decision makers, however, it is critical to have robust evidence indicating whether iSMT is as effective at reducing stress as existing group and in-person SMT programs. Currently, there is only indirect evidence that iSMT might be an adequate alternative to established in-person SMT. For example, the effect size of in-person SMT programs on stress was Cohen’s d = .73 in a meta-analysis published by Richardson and Rothstein (2008). Meanwhile, in a recent meta-analysis on Internet-based interventions for employees, Hedges’ effect size g was .76 for guided interventions [20]. This said, there was significant heterogeneity in the two meta-analyses. To this end, we consider it particularly important to compare the effectiveness of these two different types of intervention directly in the same study.

Few randomized controlled trials have compared the effects of stress management training in offered in different forms (i.e., gSMT versus Internet- or computer-based SMT) [25, 26]. Findings to date have revealed no significant difference between these interventions, with regard to stress reduction [25, 26]. Although these studies suggest that employees benefit equally from iSMT and gSMT, such a conclusion must be considered premature. Statements about equivalence or non-inferiority require adequate sample calculations and a pre-defined margin of equivalence or non-inferiority [27]. Given non-inferiority regarding mental health benefits, it is also important to allocate limited financial resources as efficiently as possible. Therefore, trial-based economic evaluations are recommended, since comparing the costs and consequences of gSMT and iSMT can provide valuable information for policymakers [28].

In the recent years, different iSMT programs, like “GET.ON Stress”, have been implemented in routine care. This program has already been evaluated in a series of randomized controlled trials in the general working population with elevated stress, demonstrating significant effects, in terms of reducing stress and depressive symptoms, relative to be being on a waiting list [29–31]. Moreover, economic evaluation of the intervention indicates that it is likely to be of good value for the money in occupational healthcare [23]. In this trial, the iSMT “GET.ON Stress” will be compared to the established in-person gSMT intervention — “Gelassen und sicher im Stress “[Calm and safe under stress] [32, 33] — which may be the most often used stress management training program in German-speaking countries and has been demonstrated in a randomized controlled trial to be effective at reducing stress, as well [33, 34].

As for any complex intervention, beyond comparative efficacy and cost-effectiveness, it is important to obtain insights into moderators of the SMT under study [35]. While assessing the non-inferiority of iSMT, in terms of reducing stress, it would also be valuable to explore if there are indicators that identify who benefits most from which SMT format. Research on moderators of either format, however, is scarce. For group stress management training, different intervention- and person-related moderators of efficacy have already been studied (e.g., intervention length, an intervention’s number of components, industry sector) [10] without generating clear conclusions for implementation in occupational settings. On average, iSMT yields greater effects when complemented with guidance through personal contact, contrary to pure self-help interventions [19, 20] and when it takes no more than nine weeks to finish the intervention [19]. Identifying moderators for each of these formats would provide novel information for intervention developers and decision makers, both in public and occupational health, especially if non-inferiority is established. For example, one study evaluating how well different health service formats were accepted by respondents of the general population revealed that people found face-to-face counselling and therapies more useful than Internet-based counselling, and that participants reported greater intentions to use face-to-face services to address emotional problems [36]. Similarly, respondents to another survey preferred traditional face-to-face stress management over Internet-based stress management [37]. To our knowledge, no study to date has empirically investigated the characteristics of the different training formats as potential moderators of these interventions’ efficacy.

In keeping with the above issues, the primary objective of this pragmatic trial is (1) to test the hypothesis that the iSMT intervention “GET.ON Stress” [29] is non-inferior to the gSMT intervention “Gelassen und sicher im Stress – calm and safe under stress” [32, 33] in employees, in terms of reducing perceived stress. Further aims are (2) to compare the two conditions from a cost perspective, and (3) to explore moderators of this comparative efficacy.

## Methods

### Study design

The declaration of Helsinki for Ethical Principles for Medical Research Involving Human states that “The benefits, risks, burdens and effectiveness of a new intervention must be tested against those of the best proven intervention(s)” [38]. Accordingly, we will test the non-inferiority and cost-effectiveness of the relatively new iSMT intervention “GET.ON Stress” [29] against the best proven and probably most adopted intervention for stress management in Germany speaking countries, that is the gSMT intervention “Gelassen und sicher im Stress – calm and safe under stress” [32, 33]. In a randomized controlled trial, one group of participants will receive access to iSMT, whereas another group will participate in gSMT.”

Outcome assessments will take place at baseline (T1), and both three months (T3) and six months (T4) after allocation to the study conditions. To mimic real-life occupational practice, participants in iSMT will be granted access to the intervention immediately after randomization, whereas participants in gSMT will have to wait until a sufficiently large number of participants has been recruited and randomized into this condition to fill group sessions. For this reason, participants in each condition will also need to attend in a short extra assessment (T2) right before their first training session.

The primary outcome will be the level of perceived stress at T3. There will be no restrictions to the use of other care as usual (CAU). This study protocol describes the design of the pragmatic trial based on Standard Protocol Items: Recommendations for Interventional Trials (SPIRIT) guidelines [39]. The study also will be conducted in accordance with the Consolidated Standards of Reporting Non-inferiority Trials (CONSORT) [27].

### Participants

The target group will consist of workers from small- and medium-sized enterprises. For pragmatic reasons, the study will take place in the Rhine-Ruhr metropolitan region in Germany, which has a population of approximately 10 million people. Participants must a) be employed, b) have access to the Internet to complete online training sessions weekly, c) be able to visit gSMT sessions in their local area, d) give their informed consent to participate, and e) complete the baseline assessment (T1). Referring to national guidelines for prevention, we keep the exclusion criteria at a minimum. Applicants a) who are participating in another stress management training at the time of study registration will be excluded. To ensure participants’ safety, those b) with an elevated risk of suicide, indicated as a score > 1 on item 9 of the Beck Depression Inventory [40], will be also excluded and receive an email containing information about how to obtain adequate help and listing the telephone numbers of relevant services.

### Recruitment and procedures

**Fig. 1:**
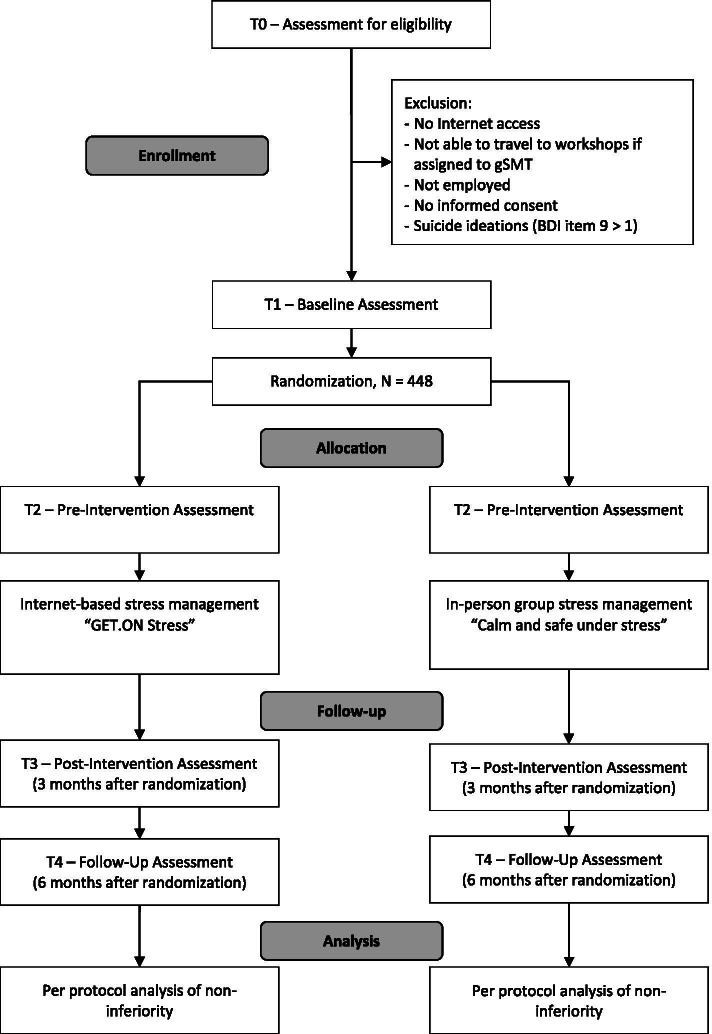
Study Flow

Recruitment will occur in the winter through summer of 2022, employing two major strategies. To attract interested organizations, we will use different media channels to motivate owners of small- and medium-sized companies (staff headcount < 250) in the target region to offer the intervention to their workforce. To attract individuals, health insurance companies will advertise the study via announcements in print membership magazines and media channels (e.g., Facebook, Twitter). Registration into the study will take place at an individual level, so employees need not self-disclose to their employer that they want to participate. An open-access website will provide information on study conditions. Applicants will sign up by providing an email address and name or pseudonym on the website. Applicants who fulfill all inclusion criteria assessed by an online screening questionnaire, return their informed consent, and complete the baseline assessment (T1) will receive a phone call by a research assistant to inform them about further requirements for inclusion (e.g., time scheduling for each of the interventions) and to answer the applicants’ questions. Thereafter, each applicant will be randomly allocated to one of the study conditions (Fig. 1), with randomization stratified by whether or not employees work in companies concurrently conducting organizational mental health programs. Allocation concealment will be ensured by an independent researcher not otherwise involved in this study, who will randomize study participants with a separate randomization sequence for each strata (concurrent organizational health program = yes/no). We will use a restricted randomization procedure using varying block sizes of 4 to 6 generated by the software RandList (randomization.eu). This researcher will send randomization results to the study administration responsible for subject allocation. For practical reasons, blinding of participants will not be possible.

After they are informed about the outcome of randomization, participants offered iSMT will receive immediate access to the program. Participants allocated to gSMT will receive information about the times and locations of group sessions; they then will need to select in which workshop they want to participate. Just before their first training session, all participants will need to complete a short pre-intervention assessment (T2). Participants will receive up to three automated e-mail reminders if they did not attend the assessments. All assessment and Internet-based training data will be collected using a secure assessment and training system (AES, 256-bit encrypted). Both the web-based assessment system and training platform are physically located on servers belonging to Leuphana University of Lueneburg. The principal investigator DL and the first author LB will have full access to the final dataset. Two researchers will assure data integrity, independently.

### Intervention conditions

#### Internet-based stress management training

The iSMT program “GET.ON Stress” [29, 41] was designed to enhance two strategies of stress coping: problem solving [42] and emotion regulation [43] (Table 1). The intervention consists of seven modules that participants should work on following a weekly schedule. Each module consists of general information; interactive exercises; prototype training participants – so called *personas* – who represent different stressed employee groups; quizzes; audio and video files; and downloadable work sheets. In addition, at the end of sessions 2 to 6, users can choose to attain extra information and perform short exercises about the following common stress-related topics: time management, rumination and worrying, psychological detachment from work, sleep hygiene, sleeping habit rhythm and regularity, nutrition and exercise, organization of breaks during work, and social support [29, 41]. For this trial, we adapted GET.ON Stress to employees working at small- to medium-size enterprises, the adaptation pertaining to the personas within the online program who guide participants through the program. Personas are a well-established element of user-centered design in software engineering [44] that has been also used to tailor Internet-based interventions to specific target groups [45, 46]. Within GET.ON Stress, the personas fulfill several functions, following the Efficiency Model of Support [47]. The integration of personas aims to increase user engagement in the iSMT program, providing knowledge about how to complete the exercises within the program and helping users to transfer what they learn from the exercises into their daily lives; for instance, by giving examples of how employees working under similar circumstances apply a given problem-solving strategy in their daily life. In addition to program content, participants will receive written feedback from an e-coach on their exercises after each of training module, the feedback provided in accordance with the training manual. E-coaches will be psychotherapists or master’s degree-level psychologists. Based upon our experiences from previous studies, we anticipate that the e-coaches will spend roughly 30 min per feedback. To improve participants’ adherence with the intervention, the e-coaches will send reminders to participants any time they fail to complete a training module within seven days. All communication between the participant and the e-coach will take place in a secured, web-based, open-source platform, located at the Leuphana University of Lueneburg.

**Table 1:** Content of the Internet-based intervention

Session^a^	Intervention content
1	Psychoeducation on stress and coping competencies Enhancement of pleasant activities
2	Problem-solving I – identifying and differentiating solvable and unsolvable problems; developing an initial problem-solving plan Information and exercises on selected topics, which users can self-select^b^
3	Problem solving II – self-evaluating the problem-solving plan; adapting or developing a new problem-solving plan Information and exercises on selected topics, which users can self-select^b^
4	Emotion regulation I – progressive muscle relaxation Information and exercises on self-selected topics^b^
5	Emotion regulation II – acceptance and tolerance of (negative) emotions Information and exercises on self-selected topics^b^
6	Emotion regulation III – effective self-support in times of stress Information and exercises on self-selected topics^b^
7	Developing a stress-coping plan for the future

Note: ^a^ each session will last approximately 45 to 90 min; ^b^ optional exercises will cover the topics of time management, rumination and worrying, psychological detachment from work, sleep hygiene, the rhythm and regularity of sleeping habits, nutrition and exercise, organization of breaks during work, and social support

#### In-person group stress management training

The gSMT program “Gelassen und sicher im Stress “[Calm and safe under stress] [32, 33] was one of the first stress management training programs developed in Germany to prevent mental disorders and somatic disease. It is based on cognitive-behavioral techniques and conducted in groups. Since its development, it has become very influential in health promotion practice and is considered standard stress-management training in Germany. The training program consists of four major modules, incorporating different strategies for coping with stress: progressive muscle relaxation; problem-solving techniques; cognitive restructuring of dysfunctional attitudes; and enhancement of pleasant activities. To mirror everyday practices using “Calm and safe under stress”, all the while optimizing the intervention’s effects, present-trial participants will take part in three training sessions, each lasting roughly three hours over seven weeks (Table 2), with a maximum of 15 participants in each group. The group sessions will take place in seminar rooms in the target region, rented by the research team. A stress management trainer will lead every training group. In each training session, the trainer will provide an introduction describing the forthcoming exercises. Within the exercises, participants will provide examples of their daily work life. Participants then will complete the exercises within the whole group, in pairs, or by themselves. All trainers will be either psychologists or psychotherapists who have participated in a five-day train-the-trainer program, held by the developer of the ‘Calm and safe under stress’ training concept. A point-by-point description of both interventions, based on the template for intervention description and replication (TIDieR) [48], can be viewed in the Supplementary Information.

**Table 2:** Content of the in-person group-based intervention

Day ^a^	Session	Intervention content
1	1	Psychoeducation regarding stress and coping competencies
	2	Progressive muscle relaxation – introduction and guided practice^b^
	3	Cognitive restructuring of dysfunctional patterns of thinking I
2	4	Enhancing pleasant activities I Cognitive restructuring of dysfunctional patterns of thinking II
	5	Problem-solving I – self-evaluating the meaning of different problems on stress; developing an initial problem-solving plan
3	6	Enhancing pleasant activities II Cognitive restructuring of dysfunctional attitudes Problem-solving II - self-evaluating the problem-solving plan; adapting or developing a new problem-solving plan
	7	Developing a personal health promoting project for the future

Note: ^a^ Each day of training will last three hours and be comprised of different thematic training sessions. For each group, all training days will take place within seven weeks; ^b^ participants will be instructed to practice the relaxation technique regularly on their own between the training days. On training days 2 and 3, they can share their experience with the technique and ask for any needed assistance

### Outcome measures

#### Primary outcome

The primary outcome will be the level of self-rated stress (Table 3), measured using the Perceived Stress Scale (PSS-10) [49], a validated measure of stress that has been used extensively during evaluations of both in-person and Internet-based stress-management interventions. The PSS-10 assesses the extent to which participants experience their lives as stressful (e.g., as overstraining, unmanageable, and/or unforeseeable over the past month). It consists of ten items, each having the following 5-point Likert scale response options of 0 = never; 1 = almost never; 2 = sometimes; 3 = fairly often; and 4 = very often (summation score range 0–40). In this trial, respondents will answer items referring only to the past week. The German version of the scale has been validated in the general population [50]. An online version of the German scale has also been validated in samples of German employees, exhibiting good reliability with McDonald’s omega (ω) = .89 and good measurement invariance [51].

**Table 3:** Outcome measures and assessment points

Outcome measures	T0	T1	T2	T3	T4
Perceived Stress Scale (PSS)	✓	✓	✓	✓	✓
Center for Epidemiological Studies on Depression Scale (CES-D)	-	✓	-	✓	-
Brief Resilience Scale (BRS)	-	✓	-	✓	-
Perceived Occupational Stress (POS)	-	✓	-	✓	-
Technology Readiness (TRI 2.0)	-	✓	-	-	-
Patient Questionnaire on Therapy Expectation and Evaluation (PATHEV) – adapted for stress management training	-	✓	-	-	-
Effort-Reward-Imbalance Questionnaire (ERI)	-	✓	-	✓	-
Job Crafting Scale (JCS) - adapted	-	✓	-	✓	-
Treatment Inventory of Costs in Psychiatric Patients (TIC-P), subscale for productivity loss	-	✓	-	-	✓
Health and Labor Questionnaire (HLQ)	-	✓	-	-	✓
Assessment of Quality of Life (AQoL-6D)	-	✓	-	-	✓
Client Satisfaction Questionnaire (CSQ/CSQ-I)	-	-	-	✓	-
Preferences for online and group stress management - self-developed	-	✓	-	✓	-
Adherence to the intervention - self-developed	-	-	-	✓	-
Beck Depression Inventory (BDI), suicide ideation item	✓	-	-	-	-
Socio-economic variables (staff headcount, including income from paid employment, history of health service use, current health-related change programs in the organization, preference for Internet-based vs. in-person group training for stress reduction)	✓	-	-	-	-

Note: T0 = Screening for eligibility; T1 = Baseline Assessment; T2 = Pre-Intervention Assessment prior to intervention beginning; T3 = Post-Intervention-Assessment, 3 months after group allocation; T4 = Post-Intervention-Assessment, 6 months after group allocation

#### Secondary outcomes

To assess negative mental health consequences of stress, reflected as depressive symptoms, we will use the 15-item version of the German adaptation of the Center for Epidemiological Studies Depression Scale (CES-D) [52, 53]. In addition, we will use the German version of the Brief Resilience Scale (BRS) [54, 55] to assess changes in participants’ ability to recover from stress. To assess work-related stress we will use the relatively new Perceived Occupational Stress scale (POS) [56], translated to German.

We will use different measures for the training evaluation. To assess user satisfaction with the intervention received, we will use the Client Satisfaction Questionnaire (CSQ) [57, 58] and the adaptation CSQ-I [59] to measure satisfaction with Internet-based interventions. Adherence to the interventions will be measured using log data from iSMT participants and self-rated questions for all participants (e.g., “How often have you tried to apply tips and techniques you have learned in your daily life?”). Within gSMT, trainers will be instructed to record participants’ attendance at every training session. We also will assess the extent to which trainers adhere to the training manual (e.g., “How well did you manage to communicate the intended training content?”).

#### Cost-effectiveness measures

To collect health service utilization and productivity losses, we will use the Treatment Inventory of Costs in Psychiatric Patients (TIC-P) [60], adapted to the German context. This questionnaire includes different single questions to estimate the impact of presenteeism on productivity losses. To estimate productivity loss due to impaired performance directly, we adapted an item extracted from the Health and Labor Questionnaire (HLQ) [61]: “If you now have to catch up on work that you have not done in the last 4 weeks due to your health problems, how many hours would you have to work?”. For cost-utility analyses, we will calculate quality-adjusted life years (QUALYs), based on the Assessment of Quality of Life (AQoL-6D) [62].

#### Potential moderators

We will explore potential moderating variables of iSMT and gSMT that might affect the comparative efficacy of the two interventions: participant age, gender, technology readiness, training experience (online vs. in-person group, yes/no), training expectations, and preferences for Internet-based or group training. To assess technology readiness, we will use the Technology Readiness Index 2.0 (TRI) [63]. We will use the Patient Questionnaire on Therapy Expectation and Evaluation (PATHEV) [64], adapted to stress-management training, to assess outcome expectancies and suitability, with regard to the training received. To explore the potential impact of workplace conditions on stress, we will use the German Version of the Effort-Reward-Imbalance Questionnaire – Short Form [65], which has reliably shown associations between work-related stress and both physical [4] and mental health [66] impairment. In addition, we will use the German version of the Job Crafting Scale, by Tims and Bakker [67, 68], which measures self-initiated changes that employees make in their own job demands and job resources to optimize their personal goals. We will use the three items with the highest item-scale-correlation for each subdomain [68], resulting in twelve questions for the whole scale. In addition, we will use self-developed questions to explore preferences for either of the training formats under study (e.g., for iSMT: “It was important, to me, that I was able to do the training at my own pace”; for gSMT: “It was important, to me, that I was in contact with other participants in one place”).

### Statistical analysis

### Sample size

We used a multi-method approach to calculate the sample size required for this trial [69]. Sample size was calculated using a non-inferiority criterion, based on previous meta-analytic evidence for the reference group (gSMT). In a systematic meta-analysis, Richardson & Rothstein identified an effect of d = 0.73 on stress reduction for occupational stress management interventions, relative to being on a waiting list [10]. This effect would correspond to 4.6 points on the PSS, assuming a standard deviation of 6.3 points, derived from normative sample data from German employees [50]. In addition, we took anchor-based clinical judgement of a meaningful difference in primary outcome measure scores into account. We conducted two focus groups with clinical (*n* = 4) and occupational health experts (n = 4). All the focus group members agreed that a reduction of at least 3 points on the PSS would display a meaningful difference for people with elevated levels of stress at baseline (PSS score ≥ 22). Finally, we set the non-inferiority margin ∆ at 2 points on the PSS at T3. Based on the predefined margin, we need 314 subjects to reject the null hypothesis of inferiority of the iSMT with 80% statistical power, assuming deterioration of not more than 2 points relative to gSMT, with two-sided α = .05. Based upon previous studies on the Internet-based intervention [29, 30], we expect that approximately 30% of participants will drop out of the study during the intervention phase (between T1 and T3). Accordingly, we aim to recruit 448 participants.

### Primary outcome analysis

Referring to the CONSORT guidelines for non-inferiority trials [27], our analysis of non-inferiority will be limited to participants who adhere to the study protocol (per-protocol analysis). That means we will only include participants who have been randomly allocated, have complete data, and attended at least five out of seven iSMT or gSMT sessions. To declare non-inferiority, the upper bound of the 95% confidence interval (CI) for the difference in mean scores on the PSS between iSMT and gSMT must be below the margin ∆. For non-inferiority, we also will conduct a separate analysis based on an intention-to-treat (ITT) sample, including all randomly-allocated subjects, to test for iSMT superiority with a one-sided alpha error set at α = .05. This procedure has been found appropriate for non-inferiority evaluations [27, 70]. For analysis of the primary outcome, we will use linear models adjusted for the baseline outcome value and stratification variable. The primary outcome will be perceived stress at T3.

### Secondary outcome analyses

Because empirical evidence on the comparative efficacy and utility of both intervention formats under investigation is scarce, we will conduct a variety of explorative analyses on secondary outcomes. We will analyze improvements in perceived stress at an individual level in both study groups, by examining the number of participants who exhibit “reliable improvement”, employing the reliable change index (RCI) proposed by Jacobson and Truax in 1991. Participants will be defined as reliably improved if their PSS-10-score declines, from T1 to T3, with a reliable change index greater than 1.96, which equals 5.8 points on the PSS-10, based on an SD_post_ = 6.3 [50] and Mc Donald’s ω = .89 [51]. Response rates will be analyzed using the Pearson χ2 statistic.

For all other continuous outcomes (Table 3), we will conduct separate linear model analyses examining for any superiority of iSMT over gSMT. Group differences in newly developed Items (e.g., user preferences towards using Internet-based vs. in-person training) will be analyzed using non-parametric statistics. In addition, for all outcomes and assessment points, Cohen’s d will be calculated to quantify the size of interventional effects by subtracting the average score of the Internet-based condition from the average score of the group condition and dividing the result by the pooled standard deviations at the corresponding assessment point.

### Economic evaluations

We will perform economic evaluations from different perspectives. From an employer’s perspective, we will conduct cost-benefit analysis (CBA), considering opportunity costs due to the time required for employees to partake of the intervention and changes in productivity loss due to reduced absenteeism and presenteeism. Since health insurance companies in several countries are legally obligated to fund preventative actions, we will conduct a separate CBA from an insurer’s perspective, including intervention costs, reflected as the common market prices for the interventions. In addition, we will conduct cost-effectiveness analysis (CEA). For the CEA, we will calculate intervention costs and costs due to productivity loss and effects of the interventions, in terms of the number of reliable improvements in perceived stress at T4. The costs and effects of both interventions will then be combined in an incremental cost-effectiveness ratio [28]. A non-parametric boot-strapping method with 95% confidence intervals will be used to account for the uncertainty of differences between the two intervention groups. Finally, we will determine the probability that the intervention is cost effective for different values of willingness-to-pay per effect unit.

### Moderator analyses

Furthermore, we will explore different moderators that might affect the comparative efficacy of iSMT vs. gSMT: patient age, gender, training experiences, technology readiness, training expectations, preferences towards in-person group- or Internet-based training, working conditions in terms of an effort-reward-imbalance, and job crafting. To explore whether any of these characteristics moderate either intervention’s effect, we will conduct separate analyses. For each potential moderator, we will add to the statistical model the potential moderator as the main effect, as well as the interaction effect between the moderator and study condition on the primary outcome.

## Discussion

The currently planned study aims to examine for non-inferiority of a guided, Internet-based stress management training (iSMT) program, relative to an established, in-person group-based, stress management training (gSMT) program, in terms of their effectiveness reducing stress in employees. To the best of our knowledge, this is the first non-inferiority trial comparing the relatively new iSMT format versus an established SMT format. Findings from the present trial will broaden evidence on SMTs in several ways.

First, there is broad evidence supporting the efficacy of both traditional occupational stress management (i.e., in-person gSMT) [9, 10, 13] and iSMT [19–22] in employees. However, data comparing the efficacy of this digital training format against an established format of stress management are limited. Previous studies performing direct comparisons [25, 26] were either not powered to demonstrate iSMT non-inferiority or equivalence, relative to gSMT, or identified no improvement with iSMT [26]. Likewise, in the field of psychotherapy, meta-analyses directly comparing these two formats revealed comparable effects on psychiatric symptomology with Internet-based and established face-to-face psychotherapy [71, 72]. Although some authors are optimistic about the comparable effect sizes of Internet interventions and face-to-face interventions, such results should be interpreted with caution. Hardly any prior study was designed to directly investigate non-inferiority or equivalence employing state-of-the-art methodology [27]. Most importantly, it seems unclear if the face-to-face comparator represents the gold-standard intervention recommended by respective national guidelines. For example, in a German-speaking sample [73], eight sessions of group therapy for depression served as a comparator. However, in Germany, outpatient therapy for depression is usually individual therapy, with a minimum of 25 sessions for CBT. Therefore, previous studies may be biased, by design, in favor of digital interventions, leading to false conclusions and offering people less-effective interventions. To overcome those shortcomings, in the present trial we will use an evidence-based and widely-applied group stress management intervention in Germany as a gold-standard comparator.

Second, the trial will conduct economic analyses comparing two stress-management formats. This is of importance for employers and policy-makers seeking to adapt strategies for occupational healthcare. Especially at small- to medium-sized companies, resources for occupational health are often limited; consequently, it is important to allocate financial resources as efficiently as possible [28]. To the best of our knowledge, this is the first trial to include an economic comparison between an Internet-based stress-management intervention and a group intervention.

Third, although non-inferiority is assumed, certain subgroups might benefit more from one stress management training format versus the other. For gSMT [10] and for iSMT [19, 20], different intervention- and person-related moderators of the intervention have already been studied. In the present trial, we will explore a variety of moderators that may guide future practices and research on selective indications for either iSMT or gSMT. The identification of format-specific moderators (e.g., preferences towards one format over another) could help employers and health service providers to offer suitable interventions for specific target groups. Likewise, person-related moderators, like technology readiness, may provide important information about the basic requirements needed before successful implementation of new technology.

In the case of non-inferiority, Internet-based stress management training is complementary to established, in-person group training in occupational settings. Furthermore, if participants in iSMT experience additional benefits from this format, relative to gSMT (e.g., in terms of cost-effectiveness, availability, flexibility of use in terms of time and/or place, other aspects of convenience), iSMT would be a promising solution, especially for those companies with few resources for occupational health promotion.

### Study limitations

Despite the potential contributions of the present study, several limitations must be considered. First, participants allocated to the Internet-based condition will be offered immediate access to the online intervention, whereas participants assigned to the in-person intervention will have to wait some time before their first workshop session. Although we will make efforts to hold the workshops promptly and to schedule these training workshops as soon as possible, we expect that people in this intervention group will start considerably later than those assigned to the Internet-based treatment group. This said, this difference between these two interventions reflects the real-life characteristics of the two training formats and contribute to the study’s external validity. Second, group allocation cannot consider participant preferences, which limits conclusions regarding the implementation and actual reach of the two formats in the general employee population [74, 75]. Due to the randomized trial design, it is difficult to compare the attractiveness of the two interventions, in terms of uptake rates. Consequently, whether the Internet-based intervention shows non-inferiority or even superiority over group-based training, it remains unclear which training format would reach more employed individuals and make a greater contribution to stress reduction in the population. In psychotherapy, surveys suggest that people prefer traditional over digital care [76], but little is known about employees’ preferences regarding SMT. To address these issues, we will measure participants’ preferences towards the two intervention formats. Insights from this trial will provide a basis for allocating resources to iSMT or gSMT and facilitate future studies offering participants the opportunity to choose their preferred format. Third, digitalization of the intervention format (Internet-based vs. in-person) may be the most obvious distinctive feature of the two interventions in the present study. However, the interventions differ in several other characteristics, like the social context (individual-based vs. group-based training), major coping strategies used (problem solving plus emotional regulation techniques vs. problem solving plus cognitive restructuring), and mode of communication (asynchronous vs. synchronous). Therefore, findings from this trial cannot be attributed solely to one interventional feature. Nonetheless, the two interventions have in common that they have already been evaluated in randomized controlled trials, are offered widely in routine practice nationally, are considered gold-standard programs for either iSMT or gSMT, and offer real-life scenarios for the delivery of Internet-based and traditional healthcare programs for stress prevention [77]. Finally, participants will experience stress elicited by a great variety of work-related stressors. The Effort-Reward-Imbalance Questionnaire as a short measure for work-related stressors is unlikely to provide a multidimensional picture of the workplace situation according to, for example, the Health and Safety Executive Management Standards [78]. This would require more detailed and substantially longer assessments. However, identifying the sources of stress in detail is a complex task and require elaborated epidemiological research that is hardly feasible to conduct alongside an intervention study.

## Conclusions

Stress reduction is a major challenge that requires a variety of evidence-based approaches to reach a greater proportion of the population. Therefore, standard formats of stress-management training should be complemented with interventions that fit society’s increasingly digital lifestyle. The current study will provide evidence about the non-inferiority, cost-effectiveness, and potential effect moderators of guided Internet-based stress management compared to in-person group-based stress management. Its findings might be an important future resource for occupational health and policy decision makers.

## Supplementary Information

**Additional file 1. **TIDieR-Checklist.

**Additional file 2. **SPIRIT-Checklist.

## Data Availability

Not applicable.

## Abbreviations

CBA: Cost-benefit analysis
CEA: Cost-effectiveness analysis
iSMT: Internet-based stress management training
ITT: Intention to treat
gSMT: Group-based stress management training
